# Relationship between night shift work, eating habits and BMI among nurses in Lebanon

**DOI:** 10.1186/s12912-020-00412-2

**Published:** 2020-04-15

**Authors:** Zeinab Samhat, Randa Attieh, Yonna Sacre

**Affiliations:** 1 Faculty of Agricultural and Food Sciences, Department of Nutrition and Dietetics, Holy Spirit University of Kaslik, P.O. Box 446, Mount -Lebanon, Jounieh, Lebanon; 2 CRED Research Centre – ESA Business School, Beirut, Lebanon

**Keywords:** Night shift work, Eating habits, Body mass index, Nurses, Lebanon

## Abstract

**Background:**

The objective of this cross-sectional study was to evaluate the relationships between night shift work, eating habits and body mass index (BMI) among Lebanese nurses.

**Methods:**

A total of 307 nurses were randomly selected from five hospitals located in Beirut. Data about demographic and professional characteristics, anthropometric measures, dietary habits and intakes were collected through a validated questionnaire. To study the relationship between night shift work, eating habits and BMI, chi-square test, t-test and logistic regressions were used.

**Results:**

The majority of nurses (78. 2%) had irregular meals timing with a significant decrease in the number of complete meals consumed during the day and an increase in the number of snacks consumed during night (*p* < 0. 05). The most consumed snacks during night shifts were sweets and potato chips. The findings highlighted that BMI and waist circumference significantly increased with the number of years of work (*r* = 0.175; *p* < 0.05) and the cumulative number of night shifts hours over the entire work history (*r* = 0.135/*p* < 0. 05).

**Conclusion:**

Night shift work is positively associated with abnormal eating patterns and BMI among Lebanese nurses. However the increase in BMI is not related to eating habits.

## Background

Night shift work is frequent in some professional sectors such as in hospitals, retail sectors, and transportation. According to the Lebanese labour code, night shift work is defined as any job performed between 8 pm and 5 am during summer and between 7 pm and 6 am during winter, at least two times per week [1]. Night shift work may negatively affect health [2, 3]. Several studies have confirmed that there is a direct relationship between night work and heart disease, altered lipid profile, weight gain, and metabolic syndrome [4–6]. Working during night affects health since it disrupts circadian rhythms and eating habits, compromise cognitive ability, cause fatigue, lack of sleep and digestive problems. These factors can lead to weight gain, obesity, cardiovascular disease, metabolic disorders, type 2 diabetes and breast cancer [7–10]. Night shift work is associated with an increase in BMI and can lead to overweight and obesity [11–16]. Cumulative night shift work is strongly related to an increase in BMI, waist circumference [17–19], hip circumference and waist to hip ratio [20]. Weight gain associated with night shift work may be the result of an excess of calorie intake and a lack of physical activity during night [21, 22]. For example, night shift nurses have irregular meals timing, snack more, have some preference for high fat and high sugar foods and do not practice any physical activities outside their occupation because of the fatigue associated with the nature of their work [23, 24, 15, 25]. The frequent consumption of high sugar and high fat foods and the lack of physical activity observed among night shift nurses can lead to an accumulation of subcutaneous fat [21, 22] with subsequent increase in body weight [25, 26]. To our knowledge, no studies have yet been published and/or carried out on night shift work and its effect on eating habits among nurses in Lebanon. Therefore, this study allowed us to add some values to the scientific literature by evaluating the impact of night shift work on the diet of Lebanese nurses and to determine if there is a possible relationship between night shift work, diet and an increase in BMI.

## Methods

### Participants selection and recruitment

This cross-sectional study was performed between January and June 2017. A total of 334 nurses working at night in different care services were randomly selected from five hospitals in Beirut. Participants were recruited with the help of the nursing director of each hospital. They were anonymously interviewed using a questionnaire. Participants were males and females, aged 24–45 years, with at least 2 years experiences and without any health problems. Twenty-seven nurses declined to participate because they did not have enough time to fill the questionnaire. Thus the final number of participants was 307 nurses. A written informed consent was obtained from the participants prior to their participation.

### Data collection

A total of 307 individual interviews were conducted using a pre-tested, face-to-face questionnaire adapted from two validated questionnaires including the Standard Shiftwork Index questionnaire [27] and the EPIC-Norfolk Food Frequency Questionnaire (FFQ) [28]. The questionnaire was completed during working hours. All nurses coming across were asked to participate to the study. Only those who had given their consent, completed the questionnaire. For confidentiality reasons all respondents have been anonymised. Information about age, gender, number of years of experience, number of night shift hours per month, level of physical activity, BMI, waist circumference, number and type of meals consumed and meal times were collected. Dietary intakes were estimated by a FFQ consisting of seven food groups and five beverage items: starch, fruits, vegetables, milk and dairy products, meat, fat, sweets, caffeinated drinks, fresh juices, commercial juices, soda and energy drinks. The categories of frequency of use were: never or rarely, once per day, two to three times per day, four to five times per day, six and more times per day [28]. The frequencies used to measure the daily intake of each food and beverage portion were: never or rarely =0, once per day = 1, two to 3 times per day = 2.5, four to five times per day = 4.5 and six and more times per day = 6.The frequencies of daily consumption were compared to the recommended frequencies set by the Lebanese Food pyramid guidelines [29]. The frequencies of daily intakes considered adequate for the Lebanese population are six servings and more for starch, 2 servings for fruits, 2–3 servings for vegetable, 2–3 serving for fat, 3 servings for milk and dairy products, 5–6 servings for meat, 3–4 cups for caffeinated drinks and never or rarely for sweets, fresh juices, commercial juices, sodas and energy drinks [29]. The degree of exposure to night shift work was determined by the number of years of experience as a night shift worker and the number of night shift hours over the entire work history. BMI and waist circumference were considered as the indicators of overweight and obesity. A BMI > 25 kg / m^2^ and a waist circumference > 88 cm in women and > 102 cm in men were considered as abnormal results [30]. The level of physical activity was evaluated as follows: “inactive” for those who do not practice any physical activity outside their occupation, “moderately active” for those who practice at least two times per week and “very active” for those who practice for more than three times per week.

### Statistical analysis

The data obtained were analyzed using the SPSS 22 statistical package. Chi-square test, Odds Ratio and logistic regressions were used to determine the relationships between night work, eating habits and the risk of obesity. T-test was used to compare the daily average consumption of the different food groups and beverage items with the daily average consumption of the same foods and beverages as recommended by the Lebanese food pyramid guideline. The threshold of significance was set at *p* < 0.05.

## Results

The demographic, occupational and behavioral characteristics of the participants are presented in Table 1.

**Table 1:** Demographic, occupational and behavioral characteristics of the participant

Characteristics	Participants (***n*** = 307)	
	n (%)	mean (SD)
**Gender**		
Male	101 (32.9)	
Female	206 (67.1)	
**Age** (years)		
24–35	232 (75.6)	31.18 (6.67)
36–45	75 (24.4)	
**Working experience**		
Mean number of years of experience		9.13 (6.06)
Mean number of night shift / month		12.61 (3.797)
**Physical activity**		
Inactive	258 (84)	
Moderately active	35 (11.4)	
Very active	14 (4.6)	
**Smoking**		
Yes	128 (41.7)	
No	179 (58.3)	

About 78.2% of nurses had an irregularity in meals timing which contributed to a significant decrease in the number of complete meals consumed during the day after a night shift work (*p* = 0.000 < 0.05), and to an increase in the number of snacks consumed during night at work (*p* = 0.015 < 0.05). Our findings highlighted that the most consumed snacks during night were sweets for 50% of nurses, potato chips for 34.4%, fruits for 11.82% and baked goods for 3.76%. While the daily intake of fruits (t = 25.244 / *p* < 0.05) and vegetables (t = 24.285 / *p* < 0.05) was significantly lower than the daily recommended amounts set by the Lebanese Food pyramid guideline, the intake of fats, sweets, commercial juices, sodas and energy drinks was significantly higher (*p* = 0.000 < 0.05) (Table 2)*.* Of those who reported drinking excess caffeinated drinks overnight, 60.9% did so in order to stay awake. In addition, our results showed that 45.3% of nurses brought with them homemade dishes for dinner while only 29.6% preferred to consume fast food.

**Table 2:** Daily mean frequencies of consumption of the different group of foods and beverages of the study population compared to the recommendation set by the Lebanese food pyramid guidelines

	Participant (***n*** = 307)			
	Daily Recommended value	mean	t	p
**Starch**	6	5.83	1.623	0.106
**Fruits**	3	1.15	25.24	0.000
**Vegetables**	3	1.25	24.285	0.000
**Milk and milk products**	3	0.95	36.359	0.000
**Meat and meat products**	5	3.62	19.195	0.000
**Fats**	3	4.96	31.008	0.000
**Desserts**	0	1.76	16.751	0.000
**Caffeinated beverages**	4	2.36	13.294	0.000
**Commercial juices**	0	0.52	9.244	0.000
**Sodas and energy drinks**	0	1	10.932	0.000

Of the total of 307 nurses, 84% did not practice any physical activity outside their work (Table 1) and 56% gained weight since the beginning of work. Our results showed that 31.6% of the study population was overweight and 19.2%, obese (Table 3).

**Table 3:** Means (SD) of anthropometric measures and prevalence of overweight/obesity in the study population

	Participants (***N*** = 307)	
	n (%)	mean (SD)
**BMI (kg/m2)**		25.53 (4.83)
Normal weight (BMI < 24, 9 Kg/m2)	151 (49.2)	
Overweight (25 < BMI < 29, 9 Kg/m2)	97 (31.6)	
Obese (BMI ≥ 30 Kg/m2)	59 (19.2)	
**Waist Circumference (cm)**		
Women		86.23 (2.97)
Normal (≤ 88 cm)	128 (62.1%)	
Overweight (≥ 88 cm)	78 (37.9%)	
Men		99, 31 (3.56)
Normal (≤ 102 cm)	60 (59.4%)	
Overweight (≥ 102 cm)	41 (40.6%)	

BMI and waist circumference in both women and men increased proportionally with the number of years of experience and the cumulative number of night shift hours over the entire work history (*p* < 0.05) (Table 4). However, no significant relationships were found between BMI / WC and the level of physical activity (*p* = 0.729 / *p* = 0.218), the number of meals consumed during the day (*p* = 0.476 / *p* = 0.572), the irregularity in meals timing (*p* = 0.684 / *p* = 0.816), the number of snacks (*p* = 0.615 / *p* = 0.256) and type of snacks (*P* = 0, 794 / *p* = 0.924) consumed during night.

**Table 4:** Association between night shift work characteristics and anthropometric measure in the study population

Night shift work characteristics	Coefficient of correlation (r) (***p*** value)		
	BMI	WC (Women)	WC (Men)
**Number of year of experience**	0.175 (0.002)	0.346(0.000)	0.178 (0.045)
**Cumulative number of night shift hours over the entire work history**	0.135 (0.018)	0.284 (0.000)	0.250 (0.012)

## Discussion

This cross-sectional study aimed to evaluate the relationship between night shift work, eating habits and BMI among nurses in Beirut. Our findings showed that night shift work affects eating habits and food choices and leads, as a result, to unhealthy eating patterns among Lebanese nurses. Similar to other authors [9, 23, 31, 32], we found that irregularity in meal timing, excessive snacking during night and consumption of high-fat and high sugar foods were common among night shift nurses. About 78.2% of nurses did not take their meals at regular times (*p* < 0.05). After their night shift, they did not take their breakfast because of a lack of appetite. They went directly to bed and had their first meal only after waking up. That irregularity in meals timing contributed to a reduction in the number of complete meals consumed during the day and to an increase in the number of snacks consumed during night. As other studies [23, 31–34], we found that the most consumed snacks during night were high fat and high sugar foods such as sweets and potato chips. Only a minority of nurses tended to snack on fruits. According to Reed [35], stress is the main reason for which night shift nurses usually crave for high fat and high sugar foods during their work.

What seems interesting in our study is that, the majority of nurses (45.3%) brought home-made meals with them for dinner while only 29.6% regularly consumed fast food. These later did so not by preference for that type of food but by obligation because they did not always manage to take foods with them at work. On one hand, our findings contradict the results of a study carried out on 395 nurses in Saudi Arabia [36] which showed that night shift nurses consume more fast food at work because they like it. On other hand, this study supports the results of other researchers who had concluded that some night workers eat fast food at night not because they like it but because it is the most common type of food easily obtained overnight [31, 34].

In addition, our results identified that 60.6% of nurses consumed caffeinated drinks during night in order to stay awake (*p* < 0.05). Reeves et al [22], in their study reported similar results. They confirmed that night shift nurses tended to consume more stimulants such as coffee and tobacco to be more active during night. However, when it comes to smoking our findings showed that night shift work is not the main reason for which some nurses smoke. In fact, on a total of 307, 128 (41.7%) nurses had started smoking long before they were hired.

Our findings showed that night shift work leads to an unbalanced diet and reduce the level of physical activity among night shift nurses. The majority of nurses (84%) did not practice any physical activity outside their occupation because they did not have enough time or because they felt too tired after their night shift (*p* < 0.05). Indeed, some authors [25, 26] explained that night shift nurses do not engage in any physical activity even when they have time to exercise because of the fatigue associated with the nature of their work.

In our study, 56% of nurses had reported weight gain since the start of their work. After taking into consideration the age and gender of the study population, we found that BMI and waist circumference increased with the number of years of work and the cumulative night shift hours over the entire work history (*p* < 0.05). Several studies showed similar results and confirmed that night shift work contributes to an increase in BMI and waist circumference and leads to weight gain [18, 13, 15, 16, 19, 14, 20]. Some authors [21, 22] had confirmed that the weight gain associated with night work was probably the consequence of an excess of calorie intake during night and a lack of physical activity. In this study, our findings have shown that night work leads to a perturbation of eating habits and to an increase in BMI which can lead to overweight and obesity. However, unlike previous researches [21, 20, 25, 22, 37], our results highlighted that the increase in BMI and the risk of overweight and obesity associated with night shift work was not related to the eating habits of nurses. The increase in BMI and waist circumference among our participants was not significantly associated with irregularity in meals timing, number of complete meals consumed during the day and number and type of snacks consumed during night (*p* > 0.05).

Nutritional interventions would certainly prevent and / or limit the physical symptoms associated with night shift work. Practical strategies allowing night shift nurses to adopt more structured and balanced diet can be put in place with the help of a nutritionist. In this case, it is first necessary to make employers understand that night shift work has harmful effects on the health of employees and then to work in collaboration with them to implement these strategies. We could then consider monitoring the nurses over a well-defined time in order to observe the effect of these interventions and strategies. Irregular meal times, snacking, lack of physical activity, and limited food supply at night are challenges that need to be improved to reduce the risk of overweight and obesity associated with night work.

## Study limitations

The sample size representative of the general population for this study was to be recruited from all hospitals localized in Beirut. Some hospitals refused to collaborate for administrative reasons and others were excluded from our study because they had a limited number of nurses. Thus only five hospitals were retained. However these five participating hospitals are among the largest hospitals in Beirut and include a significant number of nurses, so our sample size was not significantly affected. This study was a cross-sectional and not a correlational study. We were just observing that there is an association between night shift work, eating habits and BMI among Lebanese nurses. Therefore, we cannot end up with a cause and effect and conclude that night shift is the main cause of overweight and obesity among night shift nurses. Another point is that, as anthropometric measures we were able to take just the BMI and waist circumference. BMI do not distinguish between fat and lean mass and not a sufficient tool in measuring obesity.

## Conclusion

This study showed that night shift nurses have poor eating habits which lead to an imbalance in their diet. They consume more foods with high energy values but fewer nutrient dense foods. In addition, they tend to consume more snacks than complete meals and do not practice any physical activity outside their work. However, weight gain and increased BMI and waist circumference observed among night shift nurses is not due to their eating habits. It seems appropriate to carry out more advanced research in order to better understand the process by which night work leads to weight gain and to an increase in the risk of obesity.

## Data Availability

Data will not be shared. For having this data, please contact ZS at zeinabsamhat@hotmail.com

## Abbreviations

BMI: Body Mass Index
FFQ: Food Frequency Questionnaire
